# Exploiting orthologue diversity for systematic detection of gain-of-function phenotypes

**DOI:** 10.1186/1471-2164-9-254

**Published:** 2008-05-29

**Authors:** Maria Luisa Martelli, Claudio Isella, Alessia Mira, Limin Fu, Daniela Cantarella, Enzo Medico

**Affiliations:** 1 Laboratory of Functional Genomics, The Oncogenomics Center, Institute for Cancer Research and Treatment (IRCC), University of Turin Medical School, Str. Prov. 142, 10060 Candiolo, Italy

## Abstract

**Background:**

Systematic search for genes whose gain-of-function by exogenous expression confers an advantage in cell-based selective screenings is a powerful method for unbiased functional exploration of the genome, and has the potential to disclose new targets for cancer therapy. A major limit of this approach resides in the labor-intensive cloning of resistant cells, identification of the integrated genes and validation of their ability to confer a selective advantage. Moreover, the selection has to be drastic and genes conferring a limited advantage are typically missed.

**Results:**

We developed a new functional screening strategy based on transduction of mammalian cells of a given species with an expression library from another species, followed by one-shot quantitative tracing with DNA microarrays of all library-derived transcripts before and after selection. In this way, exogenous transcripts enriched after selection, and therefore likely to confer resistance, are readily detected. We transduced a retroviral cDNA expression library from mouse testis into human and canine cells, and optimized the use of commercial murine gene expression arrays for species-specific detection of library-derived transcripts. We then conducted a functional screening by growing library-transduced canine MDCK cells in suspension, to enrich for cDNAs conferring anchorage independence. Notably, these cells show partial resistance to loss of anchorage, and the selection can be of limited stringency, compromising approaches based on clonal selection or anyway requiring high stringency. Microarray analysis revealed reproducible enrichment after three weeks of growth on polyhema for seven genes, among which the Hras proto-oncogene and Sox5. When individually transduced into MDCK cells, Sox5 specifically promoted anchorage-independent growth, thereby confirming the validity and specificity of the approach.

**Conclusion:**

The procedure described here brings substantial advantages to the field of expression cloning, being faster, more systematic and more sensitive. Indeed, this strategy allowed identification and validation of genes promoting anchorage-independent growth of epithelial cells under selection conditions not amenable to conventional expression cloning.

## Background

Functional screenings based on the gain-of-function approach proved extremely valuable in the identification of novel genes involved in key processes related to cancer onset and progression, such as neoplastic transformation, resistance to apoptosis, or escape from senescence [1-3]. Identification of a gene whose expression confers neoplastic properties to normal cells, or renders cancer cells resistant to death-promoting stimuli or drugs, directly defines that gene as a potential target for novel therapeutical strategies. Screenings of this type are usually performed in mammalian cells by transducing an expression library containing full length cDNAs into a given target cell line. Then a selective stress capable of strongly reducing cell viability and proliferative potential is applied. Only cells expressing exogenous cDNAs conferring resistance to the selection will grow and form resistant colonies [4]. Then, a huge amount of work is typically required to identify the integrated cDNAs in the resistant colonies, and to verify that they effectively mediate the selective advantage. Moreover, the selection has to be drastic to avoid the emergence of spontaneously resistant colonies, which would dramatically increase the number of false hits. As a consequence, this approach does not allow identification of genes conferring a limited advantage *per se *and potentially synergizing with others. Different strategies have been developed to overcome at least in part these limitations, such as vector mobilization and re-screening [5], or gene capture by recombination [6]. These approaches reduced the amounts of false hits to be analyzed, but were still quite labor intensive and required multiple screening cycles.

**Figure 1:**
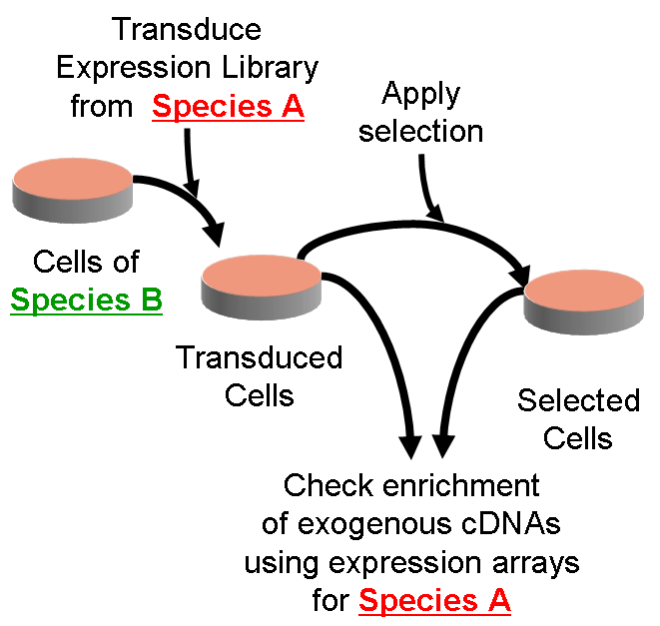
**Schematic outline of the Xenoarray analysis procedure.** An expression library from species A is transduced into target cells of species B. Efficient expression of the exogenous transcripts can be checked at this stage by comparing transduced and untransduced cells on microarrays for species A. Transduced cells are then subjected to a selective pressure to enrich for cells carrying exogenous cDNAs conferring resistance. Finally, comparing microarray signal intensities before and after selection allows simultaneous detection of all enriched exogenous transcripts, without the need of cloning resistant colonies to identify the integrated sequences.

Here we introduce a novel approach, that we named "xenoarray analysis" (Figure 1), in which standard gene expression arrays are used for tracing the abundance of exogenous cDNAs derived from the library, before and after selection, without the need of isolating clones and/or of performing multiple screening cycles. To enable specific detection of library-derived cDNAs, the species of origin of the expression library has to be different from that of the target cells. In this way, endogenous and exogenous transcripts are from different species, and sequence divergence between orthologue transcripts can be exploited as a "molecular barcode" for species-specific hybridization on microarrays. The molecular barcoding approach has been originally developed for genetic screens in yeast [7], and subsequently employed for screenings with shRNA libraries in mammalian cells [8,9]. In those cases, the bar-codes were artificial sequences whose detection required a dedicated microarray. In the present approach, the barcodes are naturally embedded in the library-derived transcripts. Therefore, commercial expression arrays could in principle be used for the task, especially because many probes are designed on the 3'-untranslated regions of the transcripts, which are particularly divergent across species [10]. To verify the potential of the approach, we assessed the species-specificity of probes contained in commercial expression arrays, defined their sensitivity on human and dog cells transduced with a mouse expression library and conducted a selective screening aimed at identifying genes rendering epithelial cells capable of growing on the absence of anchorage, a well-know feature of the neoplastic transformation process.

## Results

### Commercial gene expression arrays are species-specific

**Figure 2:**
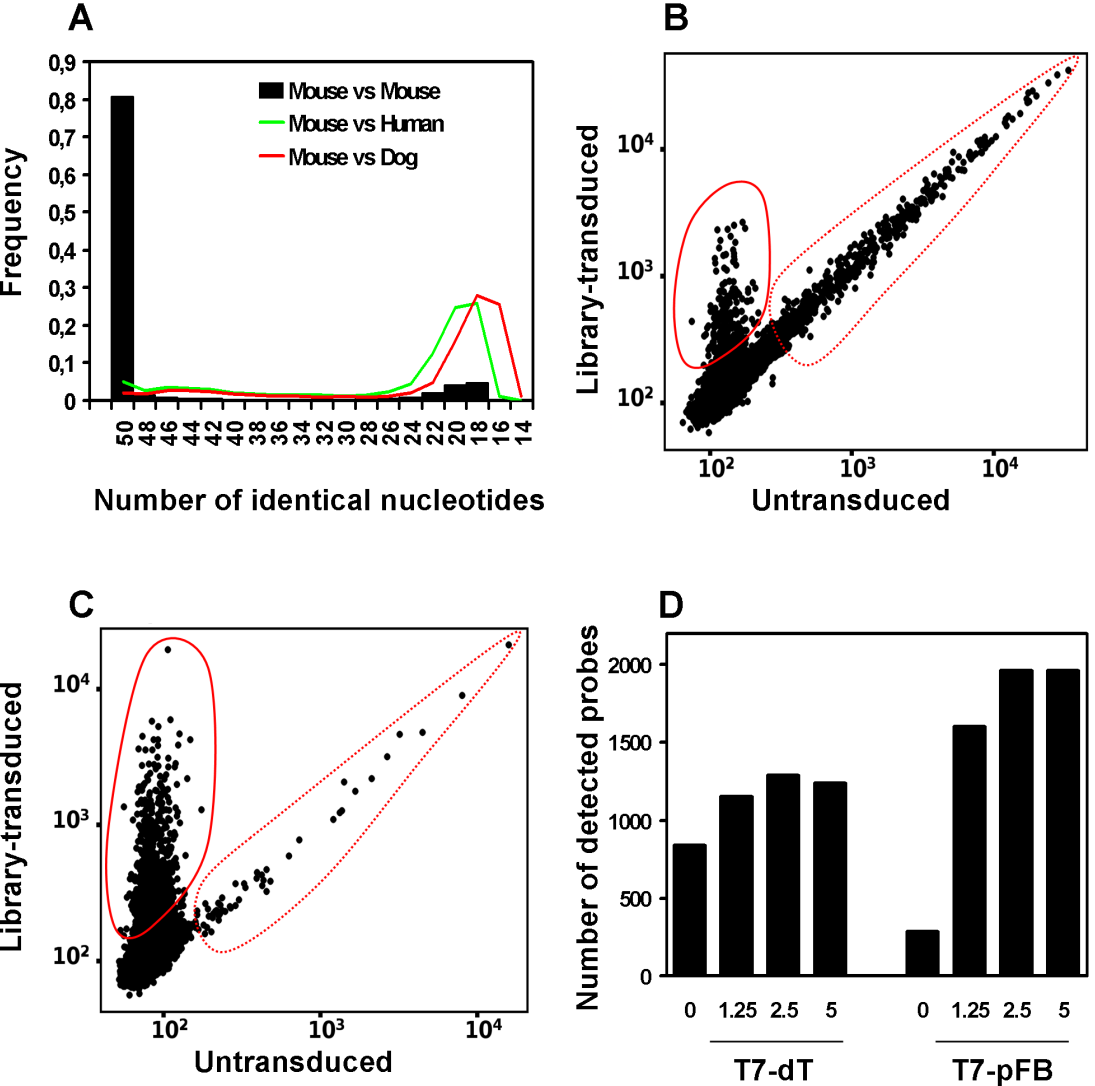
**Feasibility and setup of xenoarray analysis.** (**A**) Cross-species BLAST analysis for 50-mer probes from Illumina Mouse Whole Genome chip V1, against transcriptome databases for murine, human and canine transcripts. The best match for each probe was chosen as the BLAST hit with the highest number of identical nucleotides and the lowest e-value for significance. The histogram shows the distribution of the number of identical nucleotides between the probes and their best hits, respectively in the Murine, Canine and Human transcriptomes, as indicated. (**B, C**) Xenoarray analysis on untransduced and library-transduced HeLa cells (MOI ~1.25) using the T7-dT primer (**B**) or the T7-pFB primer (**C**). In these dot plots, each dot represents a probe signal, the coordinates of which are given by the intensity in the untransduced (*x*-axis) and in the transduced (*y*-axis) cell samples. Murine transcripts, specifically detected by the murine microarray only in the transduced cells, are highlighted by a continuous red circles. Endogenous human transcripts, giving cross-hybridization signals in both samples, are highlighted by a dotted red circle. (**D**) Numbers of probes giving significant signal using T7-dT or T7-pFB primers for Xenoarray analysis on HeLa cells transduced with the retroviral library at different MOI, from 0 (CTRL) to 5, as indicated.

To assess whether commercial expression arrays can be used for species-specific detection of exogenous transcripts within a background of endogenous transcripts, we analyzed the 46133 50-mer probes of Mouse-6_V1 expression arrays from Illumina. We noticed that 18019 out of 32826 annotated probes (55%) hybridize with at least 25 bases to the 3'-UTR or 5'-UTR regions of the corresponding mouse transcripts. Given the higher level of interspecies sequence variation in the UTR regions [10], such probes are expected to be species-specific. To further extend the analysis, each probe was blasted against the mouse, human and dog transcriptome, to check for potentially cross-species hybridizing probes (Figure 2A). According to this analysis, only a small fraction (about 5%) of the mouse array probes is likely to efficiently hybridize to the corresponding human or dog transcripts. As a control of the coverage, over 80% of the murine probes found a perfect match in the mouse transcriptome.

Therefore, the commercial arrays analyzed can in principle be used for Xenoarray analysis, without the need of a specific design. To facilitate identification and filtering of probes giving cross-hybridization signals, and subsequent assessment of exogenous transcript enrichment after the selection, we implemented a software named Xenoarray Analysis Studio (XAS). XAS enables reading and displaying gene expression data from wild-type and transduced cells, before and after selection, filtering non-specific probes, and comparing selected and unselected cells to identify enriched exogenous transcripts.

### Setup of Xenoarray analysis

To set-up the procedure, we transduced human HeLa cells with a retroviral mouse testis expression library at the estimated multiplicities of infection (MOIs) of ~1.25, ~2.5 and ~5 (for assessment of transduction efficiency, see Materials and Methods). The Scatter Plot in Figure 2B illustrates the results of gene expression profiling on control and transduced cells at MOI ~1.25, using Illumina Mouse_Ref-8_V1 arrays, and following the standard procedure with a higher amount of RNA (1 μg) and double reagents and reaction volume. Clearly the amount of exogenous transcripts specifically detected (less than 400) was not adequate for a systematic screening and required improvement. To increase detection of exogenous transcripts and decrease cross-hybridization with endogenous ones, we modified the procedure by introducing a vector-specific primer for reverse transcription (T7-pFB) and optimizing all the subsequent steps (detailed in Methods). The results obtained with this optimized protocol at the same MOI are shown in Figure 2C. The number of probes giving significant signal (detection > 0.99) in transduced cells was 1605. Conversely, only 285 probes gave significant signal in untransduced cells, indicating that over 1300 exogenous transcripts were specifically detected in transduced cells. The low background is due not only to the use of the T7-pFB primer, but also to the fact that non-specific reverse transcription of endogenous human RNAs by the pFB primer produces human cRNAs that do not hybridize efficiently to the murine array. Indeed, when the T7-pFB primer was used to reverse-transcribe a human RNA from wild-type cells and the corresponding cRNA was hybridized on human arrays, the background raised from less than 300 probes to over 1600 probes (data not shown).

To evaluate whether a higher MOI could increase detection of library-derived genes, Xenoarray analysis was performed also on the populations transduced at MOI ~2.5 and ~5, using the standard or the T7-pFB primer for cDNA synthesis. The analysis confirmed that use of the T7-pFB primer increased detection of library-derived transcripts also at higher MOIs (Figure 2D). Notably, the number of library-derived transcripts reached a plateau at MOI ~2.5 for both primers. Specific detection of almost 1700 murine transcripts at this MOI further confirmed the efficiency of retroviral expression libraries as a tool for functional screenings [2]. In view of the fact that, by average, 9000–10000 transcripts are typically detected in a microarray experiment with Illumina beadchips, we estimated that Xenoarray analysis detects the 15–20% most represented exogenous cDNAs. Rarer cDNAs are not detected in the initial transduced population, but may become detectable if enriched by the selection procedure. To estimate the enrichment rate required to render a rare cDNA detectable after selection, we exploited a key feature of gene profiling based on Serial Analysis of Gene Expression (SAGE), i.e. the absolute abundance of each transcripts measured as parts per million (PPM) [11]. In particular, we analyzed the distribution of the abundance of transcripts available from a published SAGE analysis on mouse testis [12]. We observed that, like a typical microarray experiment, SAGE identified around 10000 transcripts, and the 1700 most abundant ones had a representation of 76 PPM or higher, while the remaining transcripts had a representation of 8 to 76 PPM [see Additional file 1]. Based on this analysis, a 10-fold enrichment of a rare transcript should be enough to render it detectable by Xenoarray analysis.

### Xenoarray analysis identifies genes promoting anchorage-independent growth

**Figure 3:**
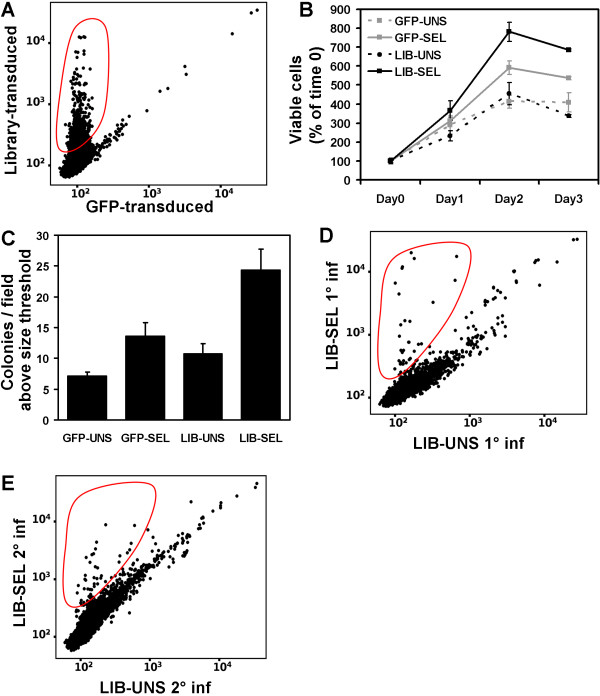
**Xenoarray analysis on MDCK cells.** (**A**) Dot plot comparing the signal intensity for GFP-transduced and library-transduced cells, as indicated. Murine transcripts, specifically detected by the murine microarray only in the transduced cells, are highlighted by a continuous red circles. (**B**) Growth curve on control and selected populations. The *x*-axis shows the time, the *y*-axis the percent of viable cells number, compared to 100% at day 0. The data represent the mean and standard deviation of triplicate values. (**C**) Soft agar growth on control and selected populations. Segmentation of single colonies and analysis of the relative diameters were performed by the Attovision 1.5 software (BD biosciences) on images captured by a BD Pathway system (BD biosciences). Briefly, a threshold of colony size was defined as the 95^th ^percentile of the colony size in pictures taken from the GFP-UNS cells. Then the number of colonies above the threshold was calculated for all fields (8 fields/sample). The bar chart indicates the average and standard error of the number of colonies/field above the threshold for the GFP- or library-transduced cells, unselected or selected on polyhema, as indicated. (**D, E**) Xenoarray analysis on library-transduced MDCK cells before (*x*-axis) and after (*y*-axis) selection, performed on first infection (**D**) and second infection (**E**). The red circles indicate the area grossly corresponding to transcripts enriched in selected cells.

HeLa cells are already transformed, highly proliferative and anchorage independent [13]. Therefore, even if their genome is remarkably stable [14], they are not optimal for selective screenings. We therefore focused on canine MDCK cells as a possible model for expression cloning of genes conferring anchorage independence. When cultured in the absence of anchorage, for instance on polyhema-coated dishes, these cells reduce their growth rate and undergo programmed cell death [15]. For the functional screening, MDCK cells were transduced at MOI ~2 with the mouse testis library or with GFP as control. Subsequently, the transduced cells were expanded, split and cultured on regular plates or selected on polyhema-coated plates, as described in Methods, to generate four populations: GFP-transduced unselected (GFP-UNS), GFP-transduced selected (GFP-SEL), library-transduced unselected (LIB-UNS) and library-transduced selected (LIB-SEL). Xenoarray analysis comparing the GFP-UNS and LIB-UNS populations resulted in the specific detection of almost 1700 library-derived transcripts in the MDCK background (Figure 3A).

The four populations were also assayed for proliferation in adherence conditions and in soft agar (Figure 3B, C). The LIB-SEL population displayed the highest growth rate in adherence, and formed much larger colonies in soft agar compared to LIB-UNS cells (p < 0.0025). A slightly increased growth was also observed for GFP-SEL compared to GFP-UNS cells (p < 0.021), possibly highlighting insertional mutagenesis events which frequently occur with retroviral vectors [16]. However, such events cannot explain the greater increase observed in the LIB-SEL population (LIB-SEL vs GFP-SEL: p < 0.02), which therefore is likely to derive from expression of advantageous exogenous transcripts. To identify these transcripts, we performed xenoarray analysis comparing LIB-SEL vs. LIB-UNS cells (Figure 3D), and observed a significant number of transcripts detected at higher levels in selected cells.

Interestingly, no library-derived transcripts are less abundant after selection. This indicates a limited stringency of the selection, allowing even rare populations to remain represented. In fact, we noticed that after an initial crisis in the first two days of culture in suspension, MDCK cells adapted to the condition by forming aggregates and reducing the proliferation rate, without massive cell death. More drastic selection procedures, such as crossing a transwell membrane and "diving" to the bottom of the well, induced loss of the majority of the library-derived transcripts (data not shown). To validate reproducibility of the selection within the same transduced population, a second selection had been conducted in parallel on the library-transduced cells described above ("Selection B"). Similarly, also the LIB-UNS population was split in two for replicate analysis (LIB-UNS A and LIB-UNS B). Also in Selection B, Xenoarray analysis highlighted enriched transcripts [see Additional file 2]. To verify if the same transcripts were enriched in both selections, we calculated the log_2 _ratio between the signals in selected and unselected cells for each transcript in each selection, and compared the results of the two selections. Over 70 percent of the genes enriched more than 2-fold in selection A were also enriched over 2-fold in selection B. To validate the 2-fold threshold for significant enrichment, we compared Xenoarray data obtained from the two LIB-UNS populations. Only two probes gave a fold-change higher than 2 (2.08 and 2.04) in this control comparison.

The 29 genes (detected by 34 probes) reproducibly enriched over 2-fold in both selections are listed in Table 1. In addition to poorly characterized genes, the screening highlighted proto-oncogenes like *Hras1, Mo*s and *Rad-9b*, known to be capable of transforming cells, conferring anchorage independence and/or promoting cell proliferation [17-19].

**Table 1:** Library-derived genes enriched in MDCK cells by two independent selections for anchorage independent growth.

Gene Symbol	Illumina Probe ID	Fold Enrichment Selection A	Fold Enrichment Selection B
Rad9b	2320064	128.77	34.88
Dhx32	2680138	103.32	49.42
Mos	5290619	55.78	12.74
Ndufv1	5420369	23.51	13.24
Hras1*	102650273	15.61	60.95
Cct2	1690113	12.13	4.90
Ldh1	2190594	10.28	8.86
Akap4	2190731	10.02	3.59
Hras1*	1980551	6.01	20.26
Mcm5	2680647	5.68	20.58
Maea	105890102	5.29	22.17
LOC331507	104760139	5.11	25.23
LOC271374	106400687	3.76	2.89
Sox6	6840717	3.64	11.39
Ncoa5	2060647	3.49	2.41
Sox5*	3190128	3.20	11.25
Temt	2320020	3.07	4.14
Fbxo6b	5690692	2.79	2.46
Sox5*	2370576	2.78	10.70
Eif2b5*	430315	2.10	2.77
Hnrpc	1570133	2.01	6.22
Eif2b5*	6900400	2.01	4.36
4933424G06Rik	1240736	145.86	66.80
5730438N18Rik	5340138	123.87	46.01
4930528F23Rik	6620368	101.24	27.16
1110003A17Rik	7000446	16.18	92.78
2700067E09Rik	3440286	11.19	48.48
8030459N02Rik	101450446	5.78	11.78
4921511C04Rik	4070411	3.55	10.61
0610007P22Rik*	130193	3.42	10.02
2500001K11Rik	5080577	3.20	9.36
0610007P22Rik*	3800433	3.16	16.36
0610007P22Rik*	6020750	2.39	13.03
2610510J17Rik	5390500	2.26	4.23

Array probes displaying increased signal in selected cells by 2-fold or higher were compared across the two selections using the XAS software. Genes appearing more than once in the table (tracked by multiple probes) are highlighted by an asterisk.

To verify the specificity of the selection hits, we carried out two alternative selection procedures, one based on serum withdrawal, and therefore not involving anchorage independence, and the other based on the crossing of a transwell membrane and "diving" to the bottom of the plate, more directly linked to anchorage independence. Then we compared the enrichment of the 34 probes emerged from polyhema selection with the enrichments observed in these two selections [see Additional file 3]. While no correlation was observed between polyhema selection and serum withdrawal (Pearson = -0.32), a striking concordance was observed between polyhema and transwell "diving" selections (Pearson = 0.94). These results confirm that cDNA enrichment is not just the result of a general tendency of a subpopulation of cells to overgrow, but rather is specifically driven by the type of selection.

Reproducible enrichment of exogenous cDNAs by parallel selection in the same transduced population does not rule out two main possible artefacts: (i) enrichment may derive from deregulation of an endogenous gene by insertional mutagenesis; (ii) a very small subpopulation of resistant cells may exist from the beginning, and a cDNA transduced in these cells may get reproducibly enriched by carryover.

Therefore, to further validate the screening hits, we performed a second, independent transduction of MDCK cells with the mouse testis library ("Infection 2"), using a lower MOI (~1). It should be noticed that double or triple infection of the same cell is not a problem for the xenoarray approach, because in the one-shot analysis the selection drivers will be present in all selected cells, while the bystanders will be diluted out. The lower MOI was chosen to reduce the possibility of phenotypes deriving from insertional mutagenesis. Again, after expansion, the cells were split to generate unselected and selected populations. Xenoarray on LIB-SEL vs LIB-UNS populations from this second infection was then compared with the results of the first infection (Figure 3E). Even with a lower MOI, Xenoarray analysis identified several enriched transcripts; those reproducibly enriched over 2-fold in both independent screenings are listed in Table 2. Notably, *Hras1 *enrichment was confirmed, albeit less evident, and the *Sox5 *and *Sox6 *transcription factors displayed the highest concordant enrichment.

**Table 2:** Library-derived genes enriched in MDCK cells by two independent infections and selections for anchorage independent growth.

Gene Symbol	Illumina Probe ID	Fold Enrichment Infection 1	Fold Enrichment Infection 2
Sox5	3190128	3.20	42.50
Sox5	2370576	2.78	23.36
Sox6	6840717	3.64	27.78
Hras1	102650273	15.61	4.25
Akap4	2190731	10.02	2.78
4921511C04Rik	4070411	3.55	2.14
Fbxo6b	5690692	2.79	2.58
Hnrpc	1570133	2.01	2.80

Array probes displaying increased signal in selected cells by 2-fold or higher were compared across the two infections using the XAS software.

Repetition of the transduction and selection procedure led to a limited validation of the hits identified in the first screening. This result corroborates the idea that process streamlining allowed by the xenoarray approach should be exploited for performing multiple independent screenings, thereby allowing identification of more consistent hits. However, it should be noted that genes enriched in only one transduction-selection experiment are not necessarily false hits. Indeed, the complexity of the integrated cDNA repertoire can vary across independent transduction experiments. This is particularly true for rare transcripts, which may not be represented in one of the two transduced populations, or may integrate in unfavourable regions of the host genome. Moreover, rare transcripts need to be highly enriched to emerge from the microarray background, and even in that case the differential signal may not necessarily be greater than 2-fold.

### Validation of the ability of SOX5 to promote anchorage-independent growth

**Figure 4:**
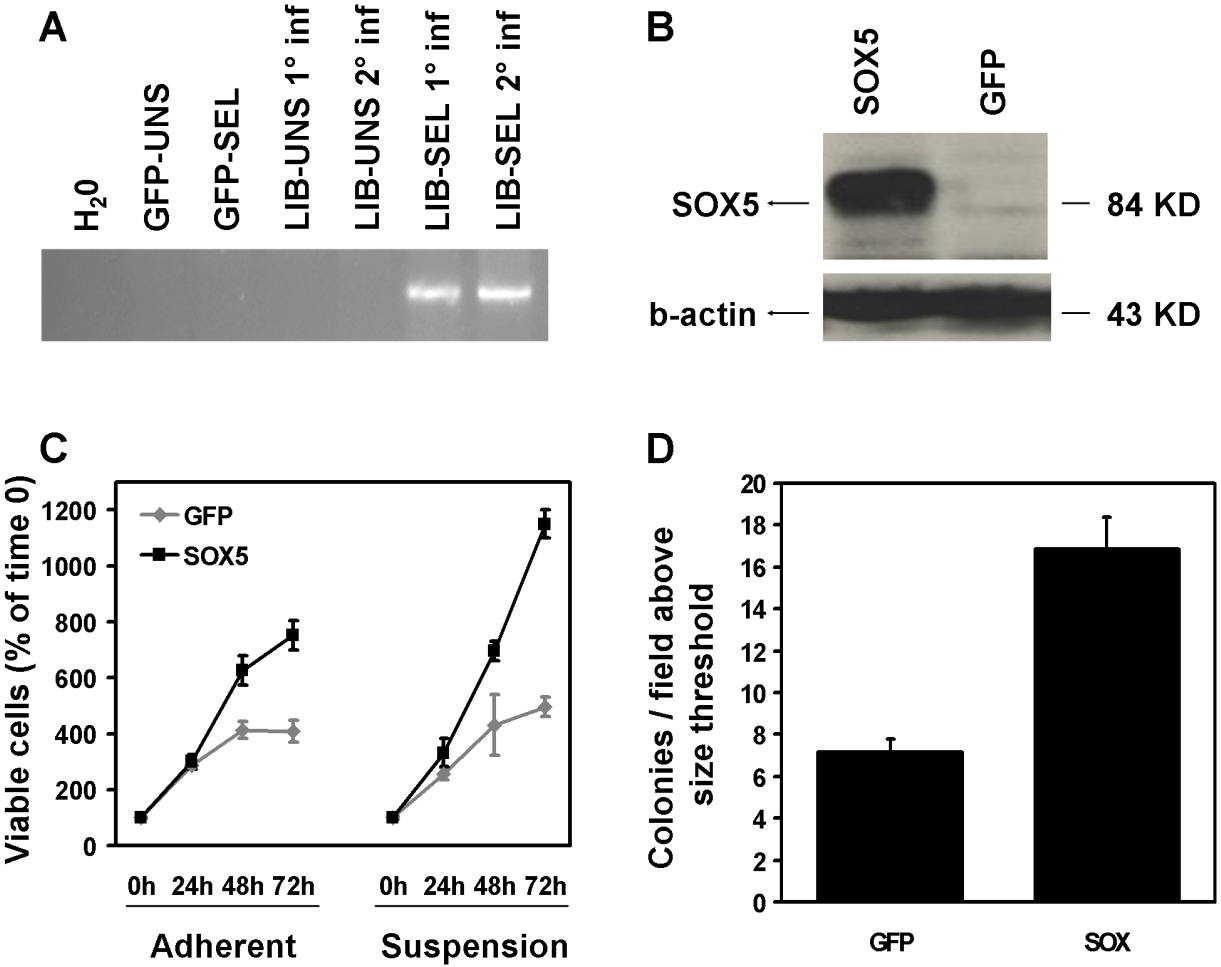
**SOX5 promotes anchorage-independent growth of MDCK cells.** (**A**) Validation of murine Sox5 enrichment in library-transduced and polyhema-selected MDCK cells. Ethidium bromide staining of PCR reactions performed on water or on cDNAs from GFP- and library-transduced cells, before and after selection, as indicated. (**B**) Western blot analysis of human Sox-5 protein expression in hSOX5-transduced MDCK cells or in GFP-transduced control cells, as indicated. Anti-β-actin was used as loading control. (**C**) Growth curve of GFP- and SOX5-transduced MDCK cells on plastic (adherent) and polyhema (suspension). The *x*-axis shows the time, the *y*-axis the percent of viable cells number, compared to 100% at day 0. The data represent the mean and standard deviation of triplicate values. (**D**) Sox5 promotes growth in soft agar. The bar chart indicates the average and standard error of the number of colonies/field above the threshold for the GFP- or SOX5-transduced cells, as indicated (8 fields/sample, p < 0.0002).

The SOX5 and SOX6 genes encode two highly similar transcription factors co-expressed in cartilage and essential for chondroblast proliferation and differentiation [20-22]. Both of them are overexpressed in gliomas [23,24], and while SOX6 was recently proposed as a possible important factor in obesity-related insulin resistance [25], SOX5 overexpression and amplification has been observed in human testicular seminoma [26]. Moreover, translocation-driven overexpression of SOX5 has recently been described in a primary splenic follicular lymphoma [27], suggesting a potentially wider role for this gene in cancer. We therefore validated murine *Sox5 *mRNA enrichment in selected MDCK cells by PCR (Figure 4A), cloned its human coding sequence into the pFB retroviral vector and transduced wild-type MDCK cells, using GFP as a control.

After confirming expression of the exogenous protein by Western Blot (Figure 4B), SOX5- or GFP-transduced populations were assayed for proliferation on plastic substrate, polyhema or soft agar (Figure 4C, D). In all three assays, increased growth was observed for SOX5-transduced cells compared to the GFP controls. Interestingly, the growth advantage was more evident when cells were cultured in the absence of anchorage (1.8-, 2.3- and 2.4- fold increase, respectively for growth on plastic, polyhema and soft agar). These data confirm that Xenoarray-based screenings identify hits with specific biological properties defined by the type of selection.

## Discussion

DNA microarrays have greatly advanced our ability to identify genes whose expression is associated with particular phenotypes or biological processes [28,29]. Nevertheless, they have been falling short in determining cause-effect relationships between genes and phenotypes, especially in the field of cancer research. Recent and more direct approaches employed microarrays for "barcode" screenings with shRNA vectors, to identify genes whose loss may render cancer cells resistant to selective stresses [8,9]. However, such genes are not immediately exploitable as therapeutical targets because their blockade is beneficial to cancer cells. Rather, they may provide the rationale for identifying other druggable genes whose loss of function is selectively toxic for cancer cells. The potential usefulness of gain-of-function-based approaches in cancer research is further confirmed by recent evidence of the involvement of the PI3K pathway in the resistance of human breast cancer to the HER2-blocking antibody Trastuzumab. In particular, Berns and colleagues identified PTEN in an shRNA-based barcode screening for resistance to Trastuzumab, and then found that the acquisition of resistance by shRNA-driven loss of PTEN is mirrored by a much stronger *in vitro *resistance phenotype driven by overexpression or mutation of PIK3CA, i.e. by a gain-of-function approach [30].

All screenings based on selection find a major challenge in the need of avoiding false hits. Being substantially streamlined, the screening described here can be easily repeated multiple times, which increases the rate of true positive hits. It should be also noticed that most of the significant enrichments observed occurred for cDNAs that were not detected in transduced cells before selection. This further confirms that the range of functional exploration extends well beyond the 1700 cDNAs already detectable in unselected cells. The xenoarray approach does not directly address a key bottleneck for expression library-based screens, i.e. their reliance on high quality, representative cDNA collections and the ability to efficiently introduce these genes into mammalian cells. However, xenoarray analysis comparing transduced and untransduced cells can be used for the optimization of library construction and transduction, allowing a one-shot measurement of the complexity of the transduced library. A specific caveat to be considered is that the xenoarray approach assumes functional conservation between orthologous proteins. However, functional conservation across mammalian species is generally the rule rather than the exception, and single hit functional conservation can be further checked using orthologous proteins databases such as P-POD [31]. Moreover, hit validation would typically imply transduction of the target cells with an ORF derived from their species of origin, or RNAi-based targeting of the endogenous gene, which would further support the biological relevance of the finding.

Our data show that one of the identified murine genes, SOX5, actually promotes anchorage-independent growth of MDCK epithelial cells also when exogenously expressed in its human version, and further support a possible role for this gene in tumor onset and progression. The third-best hit of the screening, Hras1, is a well-known proto-oncogene, and its ability to confer anchorage independence to normal cells has been well documented [32]. The most frequent mechanism of activation of RAS-family genes is point mutation [17]. However, increased expression of normal RAS proto-oncogenes due to gene amplification has also been reported to occur in human cancer [33]. Therefore, the finding of this gene as a hit can be considered as a positive control of the screening efficiency. Existing knowledge about the other screening hits is compatible with their potential role in anchorage-independent growth as well. Hnrpc encodes an mRNA-binding protein found to stabilize the mRNA and to increase expression of the urokinase receptor [34], a well-known player in cancer onset and progression [35]. The Akap4 gene is expressed only in the postmeiotic phase of spermatogenesis and its protein product anchors cAMP-dependent protein kinase A in a restricted region of the mammalian sperm flagellum [36]. Its overexpression in MDCK is therefore likely to drive PKA activation and relocalization to the cytoskeleton, thereby affecting cell motility and adhesion. However, due to its extremely restricted expression, this gene is unlikely to physiologically promote anchorage independence in epithelial cells. Fbxo6b is a member of the E3 glycoprotein-specific ubiquitin ligase family, playing a role in endoplasmic reticulum-associated degradation [37]. Its overexpression may therefore promote anchorage-independent growth by modifying the expression pattern of transmembrane glycoproteins. Given the low stringency of the polyhema growth selection on MDCK cells, which did not reduce the repertoire of library-derived transcripts detected by the xenoarray after the selection, it is likely that many of the above-described hits provide only a limited advantage, and could not be detected by a classical approach based on more drastic selection strategies. In this view, it will be interesting to assess their possible reciprocal cooperation.

The procedure described here employs full-length cDNA expression libraries derived from a given tissue or cell line, which brings advantages and disadvantages, compared to arrayed collections of open reading frames (ORFs). The main disadvantage of using libraries is that some genes may fail to be represented and others may be overrepresented. In this view, Xenoarray analysis could also be applied to ORF collections, which would allow a much tighter control on the composition and relative abundance of the cDNAs used for the screening, as well as focused exploration of gene subsets. In this case, however, it should be noted that the majority of the probes of commercial expression arrays fall outside of the transcripts' ORFs. Therefore, custom species-specific expression arrays should be designed to cover areas of non-homology within the ORF regions. As an advantage, libraries provide a more comprehensive repertoire of all the transcripts and of their isoforms expressed in the tissue of origin, thereby being more explorative. Moreover, when expression libraries are derived from cancerous tissues or cells, this approach can be combined with resequencing of the hits to highlight mutated genes, potentially exploitable as therapeutical targets. It therefore provides a powerful tool for the dissection of the mechanisms of cancer onset and progression and of resistance to anti-neoplastic treatments.

## Conclusion

The Xenoarray technology described here provides a new, efficient approach to expression cloning and functional genomics. It takes full advantage of genome-wide expression profiling to identify genes that confer resistance to a specific selective stress, thereby establishing a cause-effect relationship. Being more sensitive and systematic, the procedure does not require extreme selection stringency and isolation of resistant individual clones. In this way, also genes conferring a partial advantage can be identified and further explored for their possible reciprocal cooperation.

## Methods

### Cell Culture, Reagents and viral transduction

Madin-Darby canine kidney (MDCK) cells and HeLa cells were from ATCC. They were cultured in Dulbecco's modified Eagle's medium (DMEM) (Gibco) supplemented with 10% FBS (Sigma) in a humidified atmosphere of 5% CO2. The mouse testis retroviral expression library, packaged in the VSV envelope was purchased from Stratagene (ViraPort, Cat n. 972300). To titer the library viral supernatant, we used a GFP retroviral supernatant provided by the manufacturer at the same titer, seeding 5*10^4 ^cells onto 35 mm tissue culture plates. The following day, 1 ml of dilutions from 10^-1 ^to 10^-5 ^of the pFB-hrGFP retroviral supernatant in growth medium supplemented with 10 μg/ml DEAE-dextran (Amersham Bioscence) were added to each well. After 3 hours, an additional 1 ml of growth medium was added to each well. GFP expression analysis was performed after 48 hours by flow cytometry: cells were trypsinized, diluted in a 1% paraformalhdeide-2% FBS solution and analyzed on a FACS Calibur flow cytometer (Becton Dickinson). The titer of the library, expressed as GFP transduction units (TU) per ml, was calculated as 10^5 ^(the number of infected cells) times the fraction of green cells times the dilution factor. HeLa transduction experiments were performed by plating 5*10^4 ^cells in 35 mm wells. After one day, 1 ml of library supernatant dilutions prepared as above were added. Medium with no virus was added to generate an uninfected control. The plates were returned to 37° for 3 hours, then 1 ml of growth medium was added to each well. After 48 hours infected and control cells were expanded and used for microarray analysis. MDCK transduction was performed as above, except that 5*10^5 ^cells were infected with about 1*10^6 ^TU of viral supernatant in 60 mm dishes (MOI = 2). The pFB-hrGFP retroviral supernatant was used at the same MOI as a control. After 48 hours library- and GFP-infected cells were expanded for the functional screening.

### Genomic analysis of probes cross-hybridization

Probes cross-hybridization analysis was performed by blasting all the probes from the Illumina Mouse-6_V1 chip against the transcriptome databases from Ensembl (release 91. The interrogated databases were the mm_cdna35 and est_mus for mouse, hs_cdna36 and est_hum for human. Dog transcripts were obtained from cf_cdna_broadd1 and est_mam, which required filtering to exclude non-canine transcripts. The blastjob run was launched according to the following parameters:

*$ blastall -p blastn -i fasta -d "database" -v 100 -b 100 -o out -a 2 -W 7 -m 7*,

where the W parameter sets the alignment seed to 7 bases. The blast output data were parsed with a Perl script to display the number of identical nucleotides between each probe and its best hit.

### Anchorage-independent growth selection

Polyhema-coated 100 mm Petri dishes were prepared by applying 4 ml of a 12 mg/ml solution of poly-hydroxy-ethyl-methacrylate (polyhema; Sigma) in ethanol, drying under tissue culture hood, repeating the application once and incubating the plates overnight at 37°C. For the selection, after trypsinization, 1.5 × 10^6 ^cells were plated onto polyhema plates and cultured for one week, removing cell debris by spinning the suspension at low speed (400 rpm) and resuspending the pellet in fresh medium every 2–3 days. Cells were then allowed to recover on normal dishes for 24 hours, after which the selection was repeated for a total of 3 cycles. Selected cells were expanded on regular plates for one week before being used for microarray analysis and functional assays.

### Adherent and suspension growth assays

For cell viability assays, 10^3 ^cells of each cell line were seeded in triplicate in 96-well plates, one for each time of the growth curve assay, both on plastic and on Polyhema coated plates. The day after, a tetrazolium salt-based reagent (CellTiter96 Aqueous One Solution, Promega) was added to each well according to the instructions provided by the manufacturer. After an incubation of 2 h, absorbance was read at 490 nm on a DTX 880 plate reader (Beckman Coulter, Milan, Italy). For the soft agar growth assay, 10^4 ^cells were resuspended in 1 ml of 0.5% top agar (SeaPlaque Agarose, Cambrex, UK) in growth medium and seeded in 6-well plates previously coated with 2 ml of 1% basal agar in growth medium. The assay was performed in duplicate. After 2 weeks, phase-contrast pictures were captured and analyzed with a BD Pathway Workstation.

### RNA extraction and processing for microarray analysis

RNA was extracted using the TRIzol reagent (Invitrogen), according to the manufacturer's protocol, and then further purified using the RNeasy Mini kit from Qiagen. The quantification and quality analysis of RNA was performed on a Bioanalyzer 2100 (Agilent). Synthesis of cDNA and biotinylated cRNA was performed using the Illumina TotalPrep RNA Amplification Kit (Ambion Cat. n. IL1791), according to the manufacturer's protocol, with the following variations to optimize xenoarray analysis: (i) standard cDNA synthesis with the T7-dT(24) primer: 1 μg of total RNA was used, with the doubling of the reaction volume and of all reagents; (ii) library-specific cDNA synthesis: 20 μg of total RNA were used with 4 pm of a pFB-specific primer (T7-pFB, sequence: GGCCAGTGAATTGTAATACGACTCACTATAGGGAGGCGGCGAACCCCAGAGTCCCGCTCA, HPLC-purified from Sigma) with standard reaction conditions. The T7-pFB primer contains the T7 promoter (for cRNA synthesis) followed by a vector-specific sequence, which is present in the 3' region of all transcripts derived from the library. Quality assessment and quantification of cRNAs were performed on Bioanalyzer 2100.

Hybridization of HeLa-derived cRNAs was carried out for 18 hours on Illumina Mouse_Ref-8_V1 arrays (Cat. N. BD-26-201) according to the manufacturer's protocol, using 15 μg of T7-dT(24)-derived cRNA or 1.5 μg of T7-pFB-derived cRNA. These arrays contain circa 24000 probes exploring the transcripts contained in the Refseq database, and therefore more reliable. Hybridization of MDCK-derived cRNAs was carried out on Illumina Mouse-6_V1 arrays (Cat. N. BD-26-101), using 1.5 μg of T7-pFB-derived cRNA. These arrays contain all the RefSeq probes present in the Mouse_Ref_8_V1 arrays, plus additional 24 k probes exploring less characterized transcripts, additional Unigene clusters and singletones, to allow a more complete coverage of the transcriptome. Array washing was performed using Illumina High-stringency wash buffer for 30 min at 55°C, and followed by staining and scanning according to standard Illumina protocols. Probe intensity and detection data were obtained using the Illumina BeadStudio software, and further processed with the XAS software. Microarray data are available at GEO, dataset GSE11721.

### Xenoarray data analysis

The Xenoarray analysis pipeline was implemented in the XAS software (C++ with QT library from Trolltech [38]) and subdivided in 3 main tasks: (i) Removal of probes cross-hybridizing to endogenous transcripts. For this task, the "Detection" value provided by the Illumina Beadstudio software was used. This value ranges from 0 (non detected) to 1 (detected), with 0.99 or higher as the usual thresholds for significant detection. All probes with a Detection value higher than the threshold set by the user in untransduced cells are excluded from the subsequent steps. (ii) Data preprocessing to improve reproducibility, and reduce technical noise. The data can be either rank invariant normalized or scaled according to the tenth percentile of the average distribution of all the chips. A background reduction is then performed by subtracting 2/3 of the minimum signal value. (iii) Identification of probes giving higher signal after selection. The software compares Xenoarray data from transduced cells before and after the selection, and calculates for each probe the log_2 _of the ratio between the signals before and after selection. If multiple selection experiments are conducted, the software can compare them to identify reproducibly enriched exogenous cDNAs. The software and user guide are available on Sourceforge [39].

### Analysis of Sox5 expression by PCR and western blot

For PCR, cDNA was synthesized using the RT high capacity cDNA kit (Applied Biosystems), and the mouse-specific Sox-5 primers: 5'-GATATTGGGATCTCGCTGGA-3'; 5'- AAGTACTGCCGCATTTCCTG-3', and the following cycle program: 5' at 94°, followed by 25 cycles (2' at 94°- 30" at 55°- 30" at 72°), and 10' at 72°. For Western blot, anti-human Sox5 antibody was purchased from Abcam (Cambridge, UK). Total cellular proteins were extracted by solubilizing the cells in boiling Laemmli buffer followed by sonication. 100 μg of lysates were run on SDS-polyacrylamide gels and transferred onto nitrocellulose membranes (Hybond; GE Healthcare). Nitrocellulose-bound antibodies were detected by the ECL system (GE Healthcare).

### Sox5 cDNA cloning and expression

The full length human SOX5 cDNA was purchased from RZPD German Resource Center for Genome Research and the full coding sequence was cloned into the pFB retroviral vector (Stratagene). Retroviral supernatant was produced by transfecting pFB-SOX5 or pFB-hrGFP with the pVPack-GP (gag-pol-expressing vector; Stratagene) and the pVPack-VSV-G (env-expressing vector (Stratagene) in 293T packaging cells. The pFB-hrGFP retroviral supernatant was used as control of infection efficiency. MDCK transduction with the supernatants was performed as above.

## Abbreviations

UTR: Untranslated Regions; XAS: Xenoarray Analysis Studio; MOI: Multiplicity of Infection; SAGE: Serial Analysis of Gene Expression; PPM: Part per Million; MDCK: Madin-Darby Canine Kidney; GFP: Green Fluorescent Protein; P-POD: Princeton Protein Orthology Database; ORF: Open Reading Frame.

## Authors' contributions

EM conceived the experimental strategy. MLM and EM designed the experiments, analyzed the data and wrote the manuscript. CI performed computational analyses. AM designed and performed experiments for Sox5 expression and functional assays. LF wrote the XAS software. CI and LF contributed to the writing of the manuscript. DC performed experiments. All authors read and approved the final manuscript.

## Supplementary Material

Additional file 1 representation of mouse testis transcripts according to SAGE analysis. Transcripts are ordered by decreasing representation, measured as PPM (Parts per Million). Click here for file

Additional file 2 xenoarray analysis on library-transduced MDCK cells before and after a replicate selection performed on the first infection. Click here for file

Additional file 3 dot plots of xenoarray analysis on the 34 probes reported in Table 1. The plots compare the enrichment driven by polyhema selection (x-axis) with the enrichment driven by serum withdrawal (A) or transwell "diving" (B). Click here for file
