# Supplementary material for: Exploiting orthologue diversity for systematic detection of gain-of-function phenotypes
Source: BMC Genomics. 2008 May 29;9:254. doi: 10.1186/1471-2164-9-254 (PMC2435555; doi:10.1186/1471-2164-9-254)
Supplement: Additional file 1 — representation of mouse testis transcripts according to SAGE analysis. Transcripts are ordered by decreasing representation, measured as PPM (Parts per Million). [file 1471-2164-9-254-S1.ppt]

## Slide 1
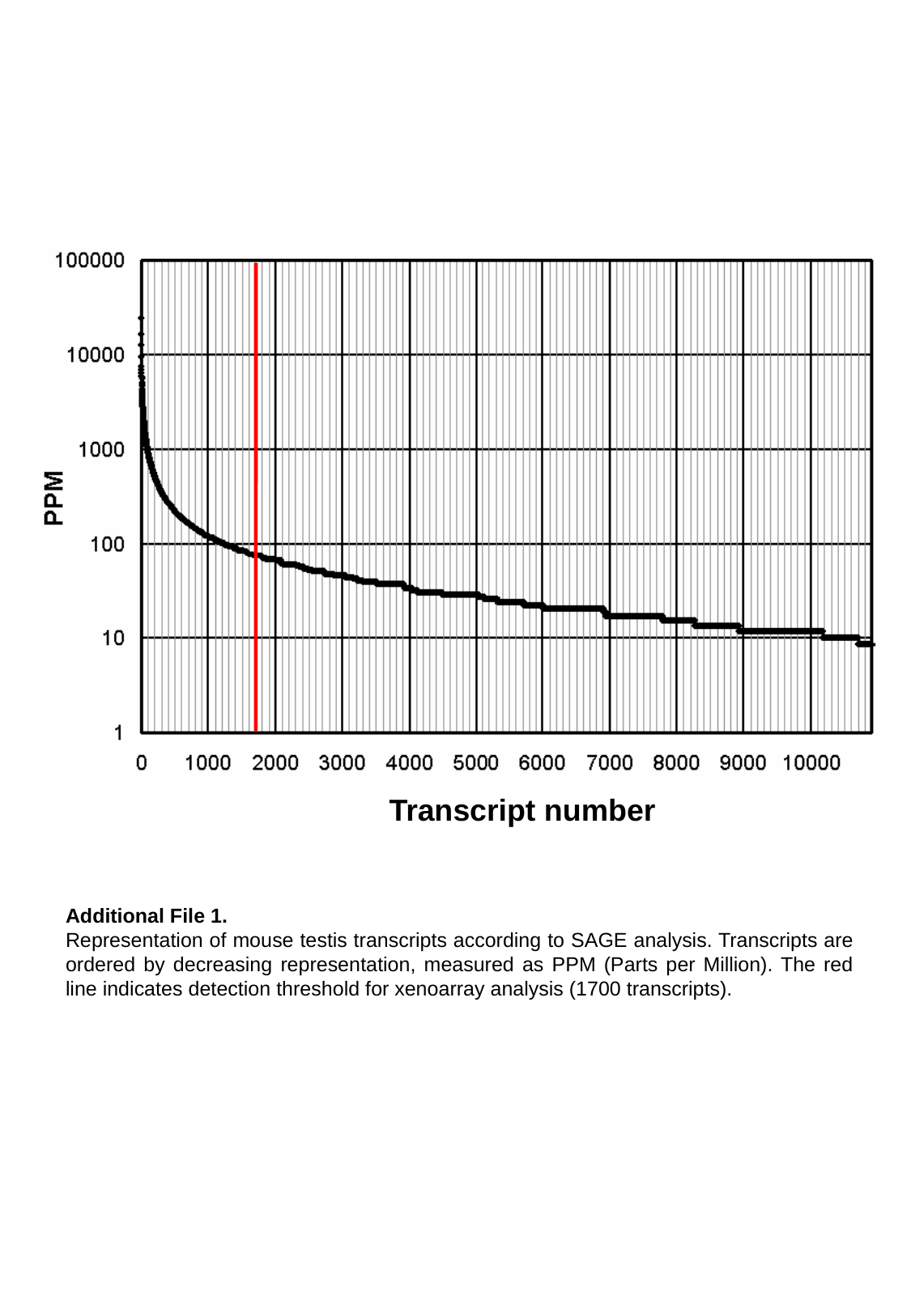

Transcript number
Additional File 1.
Representation of mouse testis transcripts according to SAGE analysis. Transcripts are ordered by decreasing representation, measured as PPM (Parts per Million). The red line indicates detection threshold for xenoarray analysis (1700 transcripts).
