# Supplementary material for: Exploiting orthologue diversity for systematic detection of gain-of-function phenotypes
Source: BMC Genomics. 2008 May 29;9:254. doi: 10.1186/1471-2164-9-254 (PMC2435555; doi:10.1186/1471-2164-9-254)
Supplement: Additional file 2 — xenoarray analysis on library-transduced MDCK cells before and after a replicate selection performed on the first infection. [file 1471-2164-9-254-S2.ppt]

## Slide 1
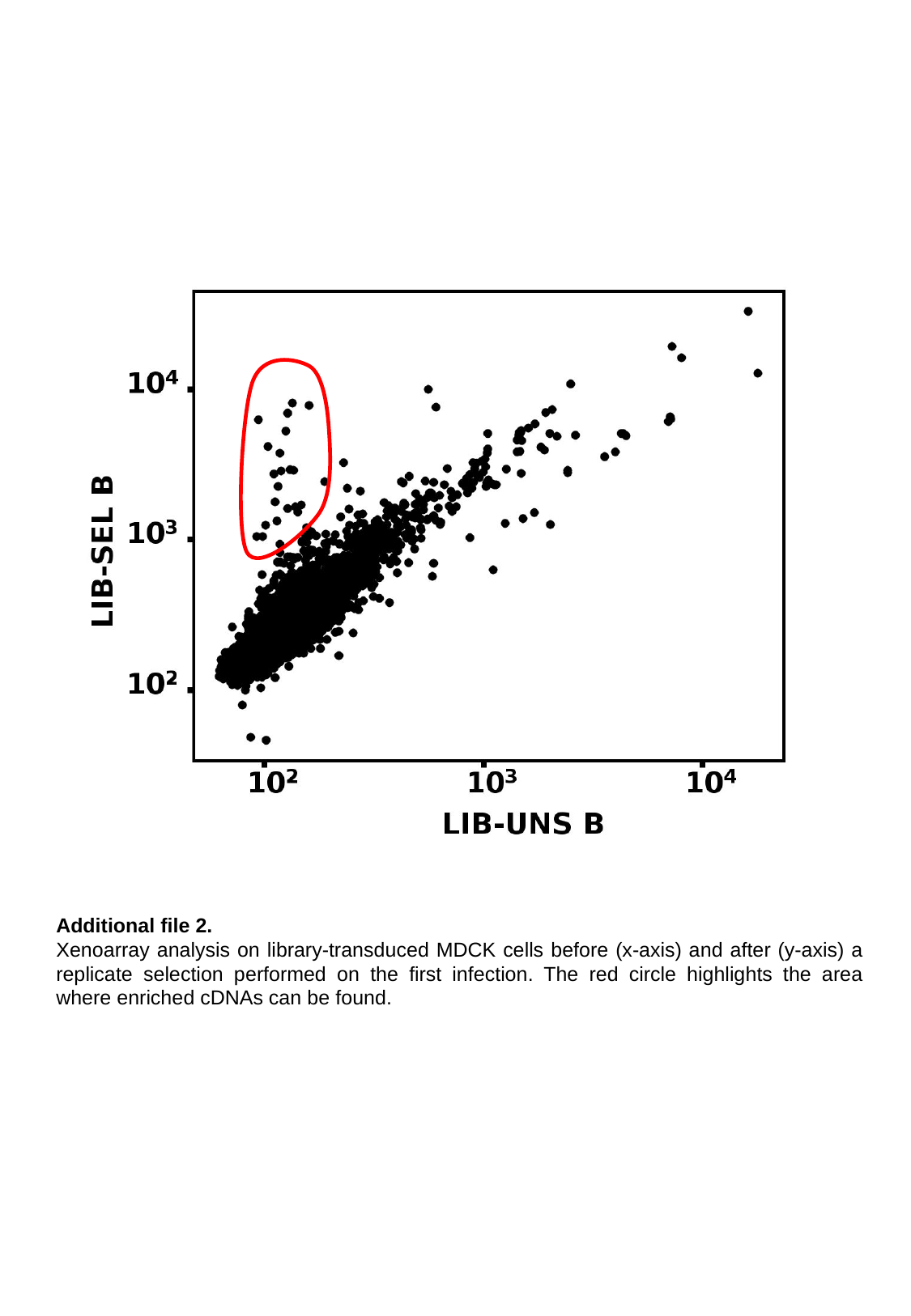

Additional file 2.
Xenoarray analysis on library-transduced MDCK cells before (x-axis) and after (y-axis) a replicate selection performed on the first infection. The red circle highlights the area where enriched cDNAs can be found.
