# Supplementary material for: Exploiting orthologue diversity for systematic detection of gain-of-function phenotypes
Source: BMC Genomics. 2008 May 29;9:254. doi: 10.1186/1471-2164-9-254 (PMC2435555; doi:10.1186/1471-2164-9-254)
Supplement: Additional file 3 — dot plots of xenoarray analysis on the 34 probes reported in Table 1. The plots compare the enrichment driven by polyhema selection (x-axis) with the enrichment driven by serum withdrawal (A) or transwell "diving" (B). [file 1471-2164-9-254-S3.ppt]

## Slide 1
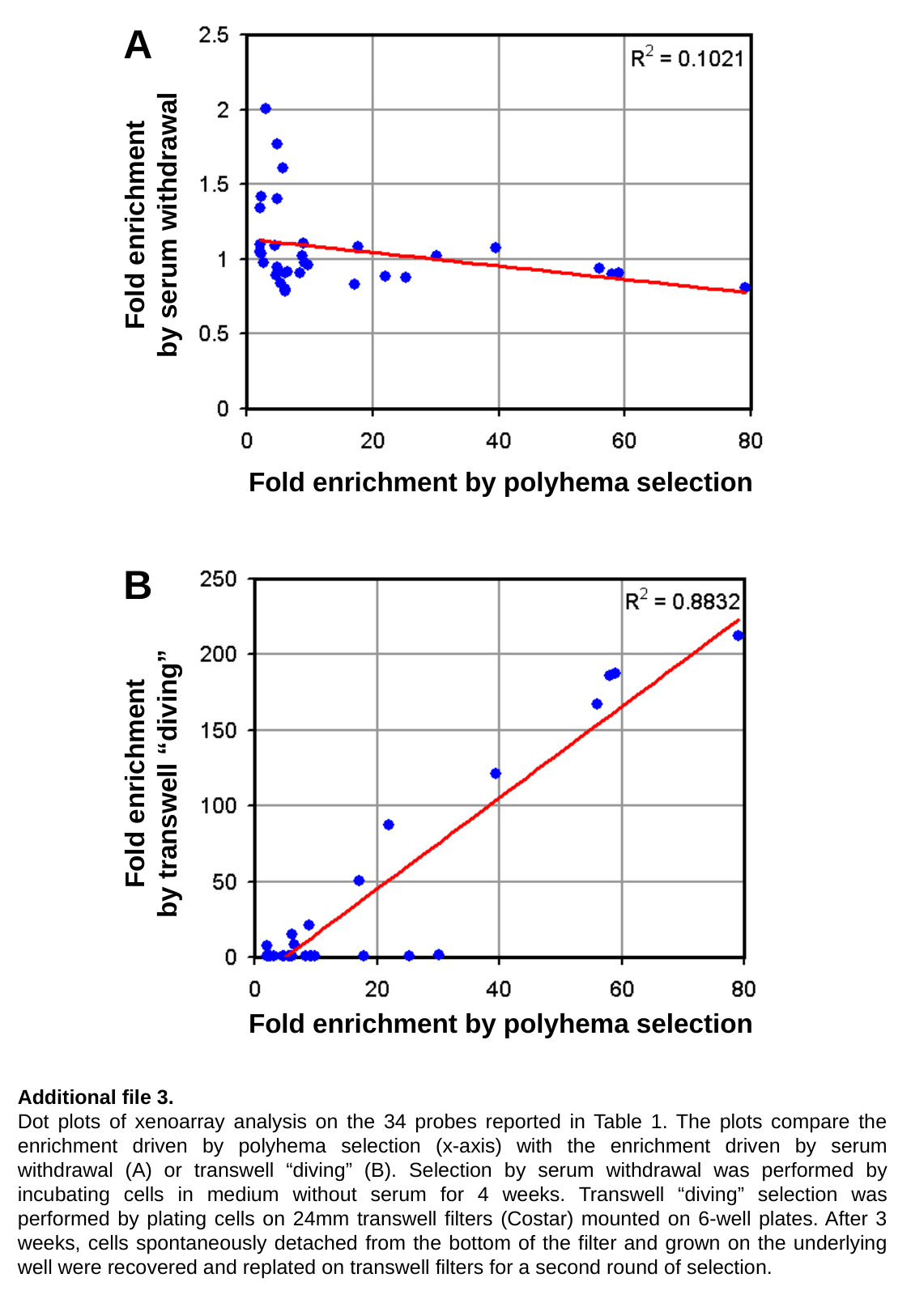

A
Fold enrichment
by serum withdrawal
Fold enrichment by polyhema selection
B
Fold enrichment
by transwell “diving”
Fold enrichment by polyhema selection
Additional file 3.
Dot plots of xenoarray analysis on the 34 probes reported in Table 1. The plots compare the enrichment driven by polyhema selection (x-axis) with the enrichment driven by serum withdrawal (A) or transwell “diving” (B). Selection by serum withdrawal was performed by incubating cells in medium without serum for 4 weeks. Transwell “diving” selection was performed by plating cells on 24mm transwell filters (Costar) mounted on 6-well plates. After 3 weeks, cells spontaneously detached from the bottom of the filter and grown on the underlying well were recovered and replated on transwell filters for a second round of selection.
